# Proposition Editorial

**Published:** 1859-12

**Authors:** 


### PROPOSITION EDITORIAL.

We wish our subscribers would "hurry up" their renewals, and busy themselves in sending additional names enough to make us rich. We had a real old fashioned cry, a boyish boo-hoo last Sunday afternoon, at Mr. Pease's Five Point Mission, with a Cincinnati "Father."

Mr. Smith was the speaker; can't say whether it was John or Joe, he only said he was Smith, and everybody knows Smith. He was not a citizen. He was describing the painful and fruitless efforts which he knew some of the fallen sons and daughters of misfortune to be making, to get employment, so that they might stand up again, and be men and women once more, but they were doomed!—no man would "hire them," and they had to sin or starve! A dreadful alternative!!

If we should be made rich by the industrious efforts of our present subscribers to increase our list, we would pay our just debts, and crow a whole year. Meanwhile, we would go to work and scatter our fortune, (as we have scattered fortunes before,) for what's the use of a fortune if you don't do something with it? The second item in the programme would be to invest money enough in the English funds to give each of our daughters a small annuity, payable quarterly, and which by no act of theirs could be transferable during their life time.

Our only boy, Robert Stephen, we would leave to hoe his own row, by the aid of a fair education and a good trade.

The third step would be to lease for a term of years, rooms of large stores and warehouses in different parts of New York, and fit them up cozily for purposes of religious worship, morning, noon and night, on Sundays. Everything should be cheerful, comfortable, and very plain, with arm chair seats for five hundred persons. There should be a prayer book with plain binding, white page, and large letters, for each seat, with a plain, common sense, pious minister, of any evangelical denomination, to officiate. In front of him there should be three men singers, and nine women singers, who should be all handsome and young, with fine and cultivated voices. No hymn should be over three verses, and no sermon over half an hour, no prayer over half a page, and that should be read. O! the profanations we have witnessed in attempts to be voluble and eloquent in prayer!—We are not an Episcopalian

These "upper room" churches should be free to every son and daughter of want, misfortune or crime. At the end of each service, a "sandwich" should be given to each person on leaving the house, or, in place of that, a Bible tract, that is, a single piece of paper with a hymn and a prayer on one side, and a scripture incident or narration, "without note or comment," on the other; besides these, arrangements should be made by which those who were really needy, should be placed in situations where they could help themselves, if they were willing to work, and willing to do their very best, for very scant wages, until time was allowed to estimate their value, for it is no charity to give a trifling person high wages; and it is a far greater charity to place it in the power of persons to earn money, than to give them money, for three times out of four, money given to the poor, is worse than money thrown away; at least it is our observation, that money gotten with only the effort of receiving it, is never duly appreciated, or wisely spent.

If any of our readers have a spare dime or dollar for the poor, they can give it to the society for the relief of "poor widows with small children;" none of the officers are paid, nor do they ever give money, nor anything else, without a personal enquiry at the place where these poor persons live, as to their character, and their habits of life; if everything is satisfactory, and they show a disposition to industry and tidiness, food or clothing is given them in small quantities at a time, and at regular short intervals.

